# Supplementary material for: A smart polymer for sequence-selective binding, pulldown, and release of DNA targets
Source: Commun Biol. 2020 Jul 10;3:369. doi: 10.1038/s42003-020-1082-2 (PMC7351716; doi:10.1038/s42003-020-1082-2)
Supplement: Supplementary file 1 — Supplementary Information [file 42003_2020_1082_MOESM1_ESM.pdf]

Supplementary Information

A smart polymer for sequence-selective binding, pulldown and release of DNA targets

Krieg et al.

Procedures

Supplementary procedure 1: MeRPy synthesis

1. Reagents

- Acrylamide-labeled anchor strand (**DNA**), stored at -20°C, protected from light
  - Stock solution 1: 2 mM in TE buffer (~7 kg/mol; ~14 µg/µL)
  - Stock solution 2: 100 µM in TE buffer (~350 g/mol; 700 ng/µL)
- Acrylamide/Sodium acrylate mixture (Label: "**A**"): Acrylamide (**AA**) mixed with Sodium acrylate (**SA**) at 99:1 mass ratio (prepare 40 wt% stock solution in the fume hood; store at 4°C protected from light)
- **TBE** buffer (5x): 500 mM Tris, 500 mM boric acid, 10 mM EDTA, pH 8.2
- **TE** buffer (100x): 1 M Tris, 100 mM EDTA, pH 8.0
- **NaCl**, 5M
- Ultra Low Range DNA ladder (**ULR**)
- Washing solution (**WS**): TE buffer (1x) containing 30 mM NaCl + 1 vol. MeOH
- Tetramethylethylenediamine (**TEMED**, stored at 4°C protected from light)
- Ammonium persulfate (**APS**) powder (stored in sealed tubes at 4°C)
- 2-ml glass vial with septum cap
- Nitrogen gas, >99.999%, passing through a Model 1000 Oxygen Trap
- Formamide-EDTA-BPB loading buffer (**FLB**): 98% Formamide(aq.) containing 5 mM EDTA, 1.7 mM Tris base and 0.02wt% BPB, pH 8.0
- Methanol (**MeOH**)

***2.* Polymerization**

A. Prepare the following solution in a septum-sealed vial (for **MeRPy-10**, see Table 1; for **MeRPy-100**, see Table 2).
   - Mix first TE, TBE, DNA and **A**
   - Degas solution #1 by bubbling with N_2 for 20 min
   - In parallel, degas ~2 ml H_2O by bubbling with N_2 for 20 min
   - Prepare fresh 10% stock solutions of TEMED and APS:
     - TEMED: 13 µL TEMED + 87 µL degassed H_2O (avoid vortexing; mix gently with pipettor)
     - APS: 100 mg APS + 1 ml degassed H_2O (avoid vortexing; mix gently with pipettor)
   - Add TEMED to sample #1, gently rock vial, then add APS while continuing N_2 bubbling

Table 1. *Synthesis of MeRPy with default anchor strand concentration (**MeRPy-10**).*

ID	TE 1x [µL]	TBE 5x [µL]	DNA 2 mM [µL]	DNA 100 µM [µL]	A 40% [µL]	TEMED 10% [µL]	APS 10wt% [µL]	Total volume [µL]
**M10**	498	160	40		101	0.4	0.4	800
**R**	70.0	20		10				100

Table 2. *Synthesis of MeRPy with 10x higher anchor strand concentration (**MeRPy-100**).*

ID	TE 1x [µL]	TBE 5x [µL]	DNA 2 mM [µL]	DNA 100 µM [µL]	A 40% [µL]	TEMED 10% [µL]	APS 10wt% [µL]	Total volume [µL]
**M100**	138.2	160	400		101	0.4	0.4	800
**R**	70.0	20		10				100

B. After adding all reagents, bubble solution gently with N_2 for another 30 min
   - The solution should become viscous during that time
C. Retract the N_2-supplying needle into the headspace of the vial, tighten screw cap, keep positive N_2 pressure in the vial.
D. Incubate sample under N_2 for at least 12 h.
E. After incubation, the solution should have turned highly viscous.
F. The vial may be opened to air.

3. Purification

A. Using a disposable syringe, take out sample #1 and mix all of it (0.8 mL) with 6.7 mL TE (1x) in a 15 ml Falcon tube.
B. Vortex for at least 10 min. Significantly longer vortexing times may be necessary if the product is highly viscous.
C. Verify that the sample is fully dispersed.
D. Rotate the sample over night in a tube rotator. (***TE_0***)
   - Note: in this protocol **red IDs** indicate that a small fraction of the solution (~50 µL) may be taken out at this point and kept for later analysis.
E. Add to the sample:
   - 25 µL NaCl (5M), if **MeRPy-10** was synthesized
   - 160 µL NaCl (5M), if **MeRPy-100** was synthesized
F. Rapidly add 5.5 mL (0.75 vol.) MeOH and vortex briefly
   - There should be no precipitate. If there's precipitate it should redisperse after further vortexing.
G. Add another 2 ml (0.25 vol.) MeOH and vortex for 20 seconds
   - A white precipitate should form immediately
H. Let sample incubate for 2 min
I. Centrifuge at 100g at r.t. for 5 min and remove supernatant (***S_1***)
   - Note: faster centrifugation will create a pellet that is difficult to redisperse
J. Remove residual supernatant droplets with a pipettor
K. Add H_2O to the pellet until the total sample volume is 7.5 ml
L. Redisperse the pellet by vortexing for 5 min
M. Add 75 µl 100x TE buffer

N. Repeat steps C–L
O. Shake sample for 30 min
P. Verify that the solution is perfectly homogeneous. If it is not, continue shaking.
Q. Split into multiple tubes and store aliquots in the -20°C freezer (**P_2**)

4. Standard analysis

A. Only for **MeRPy-100** analysis: dilute samples TE_0, S_1, and P_2 10-fold in TE buffer
B. Prepare samples for polyacrylamide gel electrophoresis (PAGE):

Lane	1	2	3	4	5	6
Sample	0.1x ULR	R	TE_0	S_1	P_2	0.1x ULR
C (DNA) max. [ng/µL]	50	70	75	37	75	50
V (sample) [µL]	4	2	2	2	2	4
V (H2O) [µL]	0	2	2	2	2	0
V (FLB) [µL]	16	16	16	16	16	16
V (loaded) [µL]	10	10	10	10	10	10

C. Run PAGE:
   - 20% Urea-PAGE
   - 0.5x TBE running buffer
   - Heat samples to 95°C for 20 seconds, then cool down to 4°C prior to loading
   - 150V, 45 min
   - Stain with SYBR Gold

D. Quantify DNA in samples R, TE_0, S_1, P_2 to determine (i) efficiency of DNA capture, (ii) efficiency of the washing step for removal of free DNA, (iii) final concentration of polymer-attached anchor strands.

Supplementary procedure 2: ssDNA pulldown and release

1. Reagents

- Methanol (**MeOH**)
- 10xTE buffer: 100 mM Tris, 10 mM EDTA, pH 8.0
- 1xTE buffer: 10 mM Tris, 1 mM EDTA, pH 8.0
- **TBE** buffer (5x): 500 mM Tris, 500 mM boric acid, 10 mM EDTA, pH 8.2
- **MeRPy-10** (0.5 wt% in TE, ~10 µM max. anchor strand concentration), stored at -20°C
- Target strand library (**TSL**): e.g, target strands #1–10 in Supplementary Table 2 (100 ng/µL each, corresponding to 1.7–16.4 µM; 63.4 µM total oligo concentration).
- Catcher strand library (**CSL**): e.g., a subset of catcher strands #1–10 in Supplementary Table 2 (100 ng/µL each, corresponding to 1.7–16.4 µM)
- Release strand library (**RSL**): e.g., mixtures of release strands #1–10 in Supplementary Table 2 (100 ng/µL each, corresponding to 1.7–16.4 µM)

- 3 M **NaCl**
- Washing solution (**WS**): TE buffer (1x) containing 30 mM NaCl + 1 vol. MeOH
- Formamide-EDTA-BPB loading buffer (**FLB**): 98% Formamide(aq.) containing 5 mM EDTA, 1.7 mM Tris base and 0.02wt% BPB, pH 8.0

2. Pulldown

A) To pull down specific targets from a **TSL**, select the appropriate **CSL** and mix together:

**MeRPy-10** [µL]	100
**TSL** [µL]	3.55
**CSL** [µL]	7.10
3M NaCl [µL]	3
Total volume [µL]	113.65

B) Vortex thoroughly, then anneal:
- 50 °C for 2 min
- Cool to 20°C with a rate of -1.5°C/min

C) Dilute sample with 114 µL 1xTE and vortex shortly

D) Add 228 µL MeOH and quickly mix sample by pipetting up and down
- There should be a precipitate

E) Centrifuge at 100 g for 5 min
- Higher centrifugal force would make the pellet difficult to re-disperse, which would prevent later release of captured targets

F) Retrieve supernatant (**S_1**)

3. Release

A) Wash pellet with 500 µL **WS** and spin again at 100 g for 5 min

B) Remove supernatant as well as possible, then add 100 µL H2O to the pellet

C) Vortex for 30 min to fully disperse the pellet

D) Add 10 µL 10xTE

E) Add 3.5 µL 3M NaCl

F) Add 5.5 µL **RSL**

G) Vortex and anneal:
- 50°C for 2 min
- Cool to 25°C with a rate of -1.5°C/min and hold at 25°C for ≥ 1h

H) Add 120 µL MeOH and quickly mix sample by pipetting up and down
- There should be a precipitate

I) Vortex, spin at 4000g for 2 min, then obtain supernatant (**S_2**)

4. Analysis

Analyze **S_1** (target-depleted **TSL**) and **S_2** (released **TSL** subset) by dPAGE
- 10% dPAGE
- Dilute 2 µL of sample with 18 µL **FLB**

- Heat samples to 95°C for 20 seconds, then cool down to 4°C prior to loading
- Stain with SYBR Gold
- 150 V, 75 min

Supplementary procedure 3: cDNA (dsDNA) pulldown

1. Reagents

- Methanol (**MeOH**)
- **TE** buffer (100x): 1 M Tris, 100 mM EDTA, pH 8.0
- **TBE** buffer (5x): 500 mM Tris, 500 mM boric acid, 10 mM EDTA, pH 8.2
- **MeRPy-100** (0.5 wt% in TE, ~100 μM max. anchor strand concentration), stored at -20°C
- Catcher strand library (**CSL**): 200 μM total oligo concentration, prepared by pooling together equal volumes of catcher strands (200 μM in TE buffer) (cf. Supplementary Table 3)
- **cDNA** (in general: **dsDNA**) samples: concentration: 10-30 ng/μL; tested size range: 150–700 bp; average size: 350–400 bp
- 2.5M **NaCl**
- Formamide-EDTA-BPB loading buffer (**FLB**): 98% Formamide(aq.) containing 5 mM EDTA, 1.7 mM Tris base and 0.02wt% BPB, pH 8.0

2. Pulldown

A. Thaw and briefly vortex an aliquot of **MeRPy-100**
B. Prepare the following sample:

MeRPy-100 [μL]	cDNA [μL]	NaCl (2.5 M) [μL]	CSL (200 μM) [μL]	Total volume [μL]
2	2	0.5	0.5	5

C. Briefly anneal the sample:
  - Quick heating to 95 °C, hold for 2 minutes
  - Quick cooling to 20 °C, hold for 5 minutes

D. Spin down
E. <u>Immediately</u> add 7.5 μl of MeOH into each solution and quickly pipet up and down to mix the sample, vortex briefly
  - Note 1: Adding the correct volume of MeOH is crucial. The volatility of MeOH may cause the solvent to drip out of the pipette. This problem is best avoided by pre-wetting the pipette tip by repeatedly aspirating and dispensing the solvent (at least 5 times) before transferring the intended volume. Alternatively, a positive displacement pipette may be used.
  - Note 2: Avoid any delay between steps C and E, as it may reduce pulldown efficiency due to re-annealing of complementary cDNA fragments.

F. Let the sample stand for 1 min
G. Centrifuge at 2000g for 1 minute
H. Verify that there's a pellet and obtain 9.5 μL of the supernatant (**SN**).

3. Analysis

A. Prepare and load following sample for denaturing PAGE (dPAGE)

SN [µL]	FLB [µL]	Total volume [µL]	Loading volume [µL]
1	9	10	9

B. Run dPAGE:
- 8% dPAGE
- 0.5x TBE running buffer
- Heat samples to 95°C for 20 seconds, then cool down to 4°C prior to loading
- 150V, 45 min
- Stain with SYBR Gold

Supplementary Notes

Supplementary Note 1: AF4

AF4 in combination with light scattering detection is a gentle separation and detection technique especially for very complex systems. The separation takes place in a channel. The application of a flow force field allows a controlled separation with reduced shear forces and interactions. The separation range of sizes is much broader (up to 1 µm) compared to routine techniques like size exclusion chromatography (SEC).[1–4] The calculation of the obtained parameters, molecular weight, radius of gyration and hydrodynamic radius allow for the estimation of the scaling properties and the apparent density of the macromolecules. Thus **MeRPy-10** shows a slightly higher scaling exponent (ν = 0.38-0.39), corresponding to a less compact conformation than **MeRPy-100** (ν = 0.32). The density calculations confirm higher density for **MeRPy-100** than for **MeRPy-10** (see Supplementary Table 1). Furthermore, the scaling exponents are typical for rather globular molecular conformation. At the same time the ratio of R_g/R_h of **MeRPY-10** is typical for coil-like structures, highly permeable by the solvent.

Supplementary Note 2: single-base-resolution pulldown efficiencies

The **relative base count** is defined as the average number of times a particular base position in a gene is counted in '+MeRPy +CSL' samples, divided by the average number of times that base position was counted in the original sample. The **relative base position** is defined as the position of a base relative to the center of CSL-targeted region on a gene. To establish a single relative base position value (rather than separate values for each isoform), the exon regions were concatenated. For this, the following transcript sequences from the Ensembl database were included: *INS*: Gene ID- 3630, Ensembl ID- ENSG00000254647, Transcript ID- INS-201, INS-202 and INS-203; *GCG*: Gene ID- 2641, Ensembl ID- ENSG00000115263l, Transcript ID- GCG-202; *TTR*: (Gene ID- 7276, Ensembl ID- ENSG00000118271, Transcript ID- TTR-201).

Supplementary Note 3: Executing the CSL generator script on Mac

- Before running the unix executable (.unk) file for the first time on a Mac, open the Terminal and type “chmod 777 ”. Note that there is a space in the end. Do not press enter yet.
- Drag and drop the .unk file into the Terminal window (or alternatively, enter the path and name of the file after “777 ”).
- Press enter.
- Now the file has execution permission.
- Execute the file directly from the Terminal by entering its path and name.
- Alternative: in the Finder, hold the control key and click on the file. Click open and open again. You may need to specify Terminal as the application to run the program.

Supplementary Figures

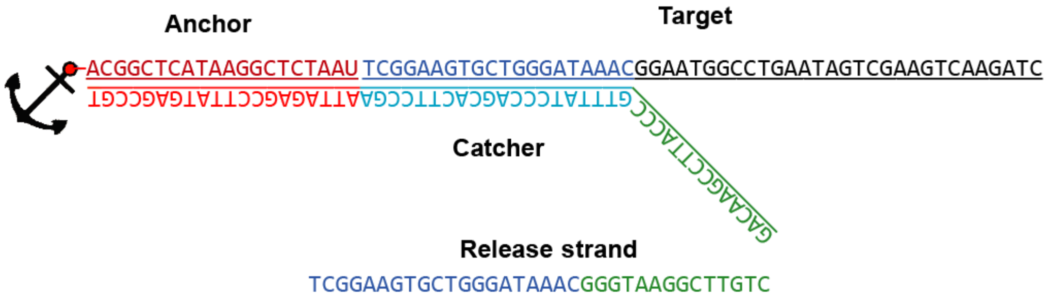

Supplementary Figure 1. *Example of an anchor-catcher-target complex and its corresponding release strand.*

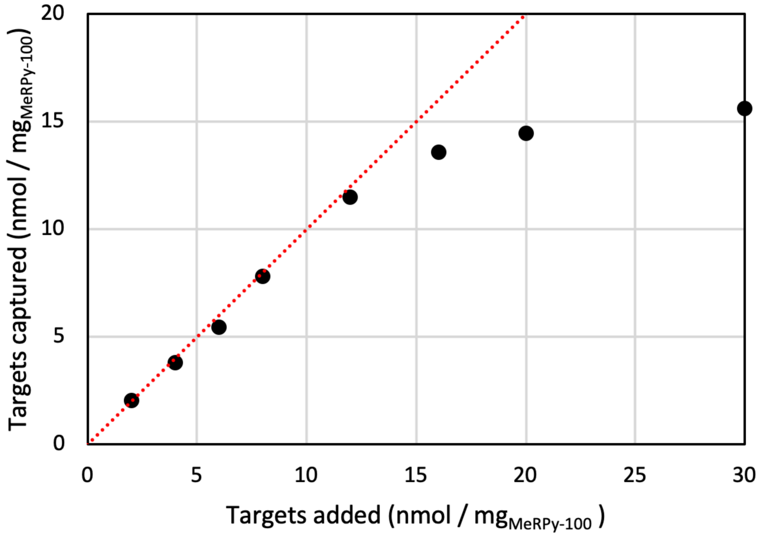

Supplementary Figure 2. *Binding of catcher strands to* ***MeRPy-100****, experimentally determined via PAGE depletion assay (black circles), and theoretical binding limit (red line).*

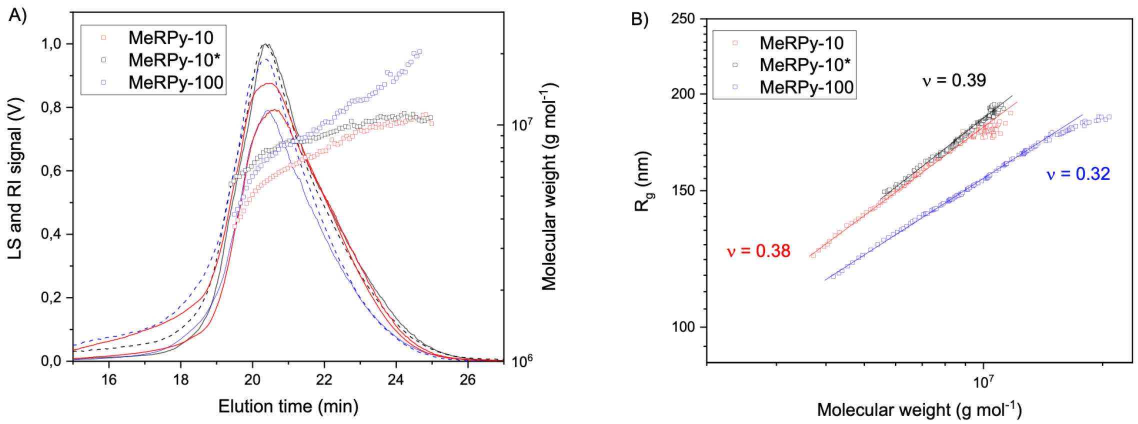

Supplementary Figure 3. *(A) AF4 fractograms: refractive index and light scattering detector signals (solid and dashed lines), molecular weights vs. elution time (squares) of the entire peak region. (B) scaling plots (R_g vs. molecular weight).*

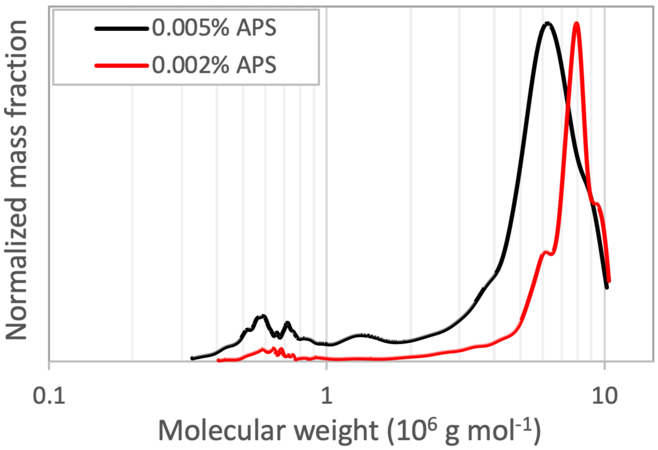

Supplementary Figure 4. *Molecular weight distributions obtained by AF4-LS. Comparison of **MeRPy-10** synthesized with 0.005wt% vs. 0.002wt% of APS.*

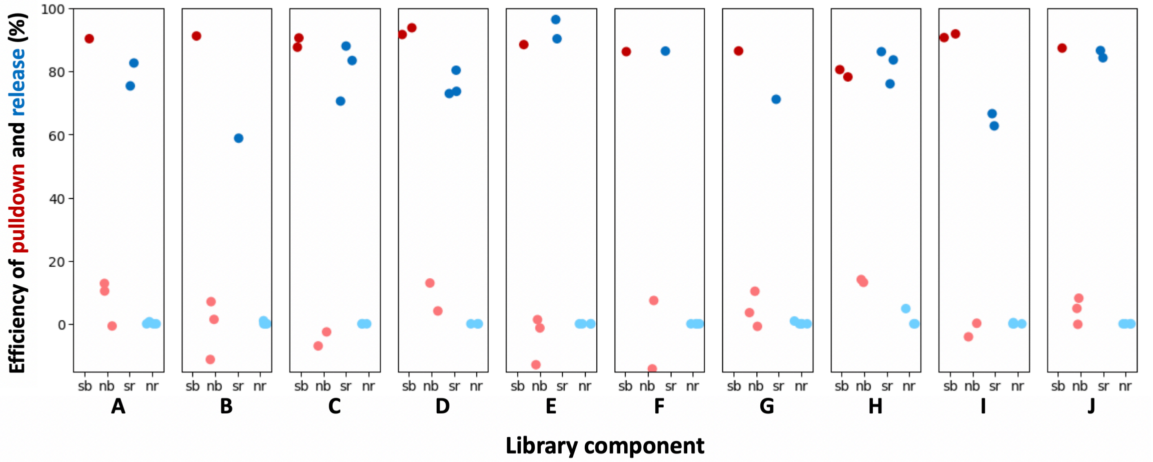

Supplementary Figure 5. *Efficiencies and specificities for individual members of the 10-component ssDNA library. sb = specific binding; nb = nonspecific binding; rs = specific release; nr = nonspecific release.*

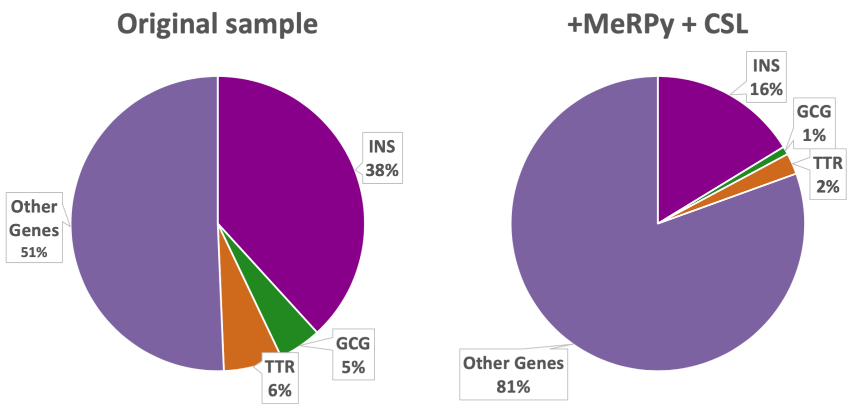

Supplementary Figure 6. *Fraction of reads consumed by INS, GCG, TTR, and other genes before (control) and after depletion with MeRPy and a combined INS-, GCG- and TTR -targeting CSL.*

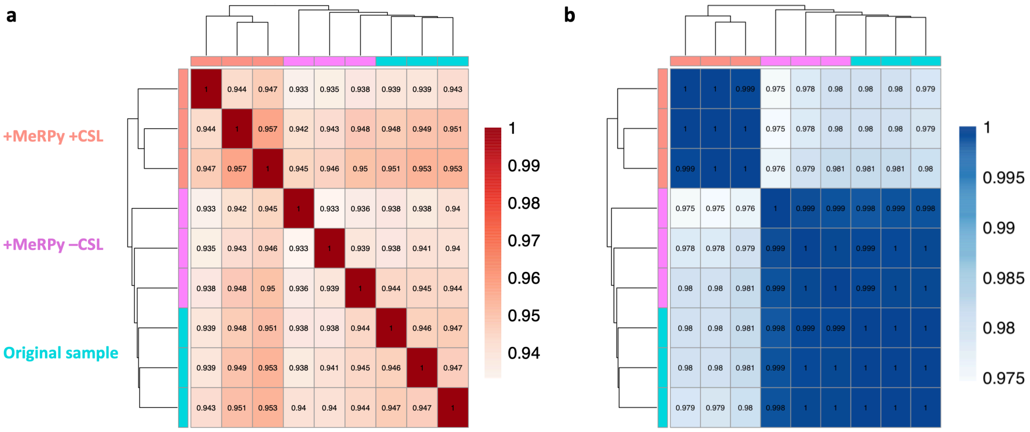

Supplementary Figure 7. **a)** Spearman and **b)** Pearson correlation (all genes, excluding depletion targets*)* for pulldown experiments targeting *INS*, *GCG* and *TTR*.

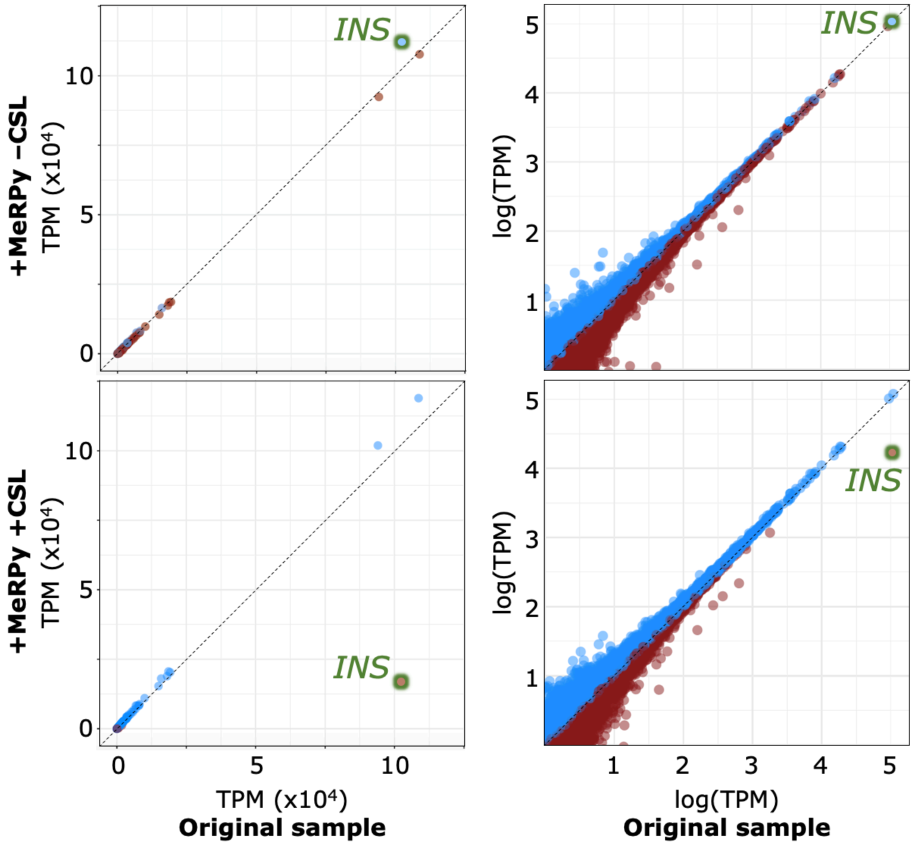

Supplementary Figure 8. *Selective depletion of high-abundance insulin (INS) cDNA from a clinical NGS library by MeRPy in presence of an INS-specific CSL. Blue and red data points represent genes that were sequenced with higher and lower number of transcripts per million (TPM), respectively, as compared to the original sample.*

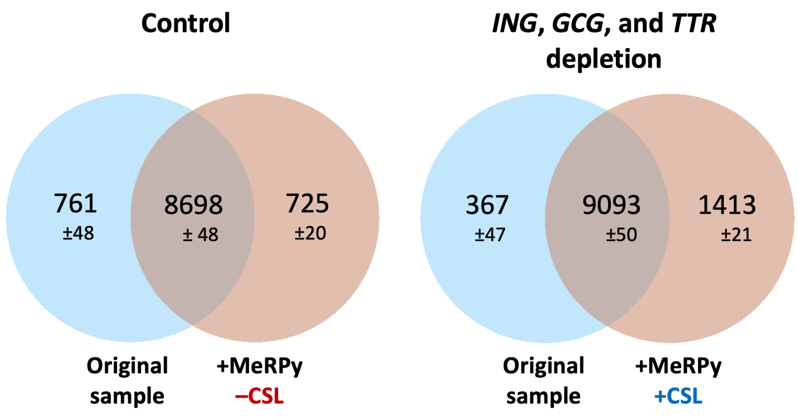

Supplementary Figure 9. *Venn Diagrams for all genes >1 TPM detection threshold in INS-, GCG-, and TTR-depleted samples (and the –CSL control) vs. the original cDNA library sample.*

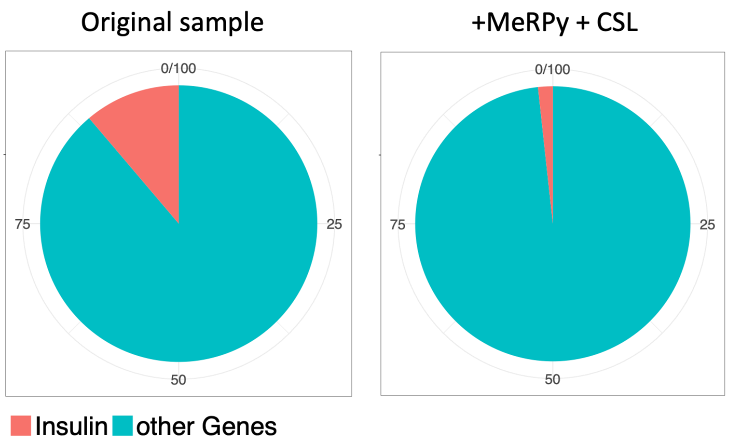

Supplementary Figure 10. Fraction of reads consumed by *INS* and other genes before (control) and after *INS* depletion with MeRPy and an *INS*-specific CSL.

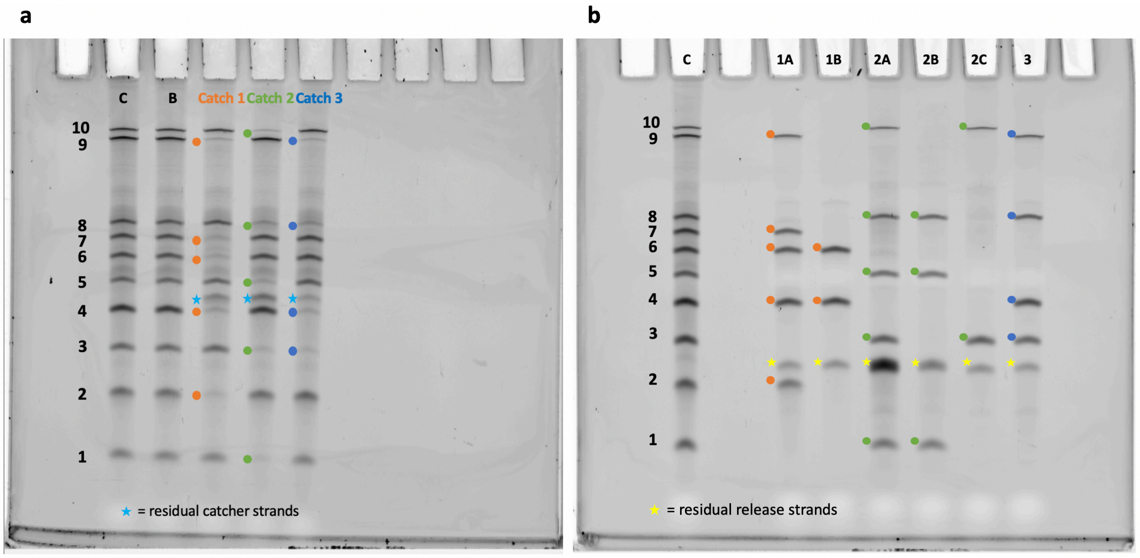

Supplementary Figure 11. *Original full-size scans of Sybr-Gold-stained dPAGE corresponding to Figure 3a. Numbers 1-10 correspond to members of the target library A-J (in reverse order).* ***a)*** *Pulldown experiment. C = control (original library). B = blank (MeRPy pulldown without catcher strands. Catch 1,2,3 = Supernatant after MeRPy pulldown with different catcher strand combinations. Selected catcher strand targets are marked with orange, green, and blue circles.* ***b)*** *Release experiment. The selected release targets are marked with orange, green, and blue circles.*

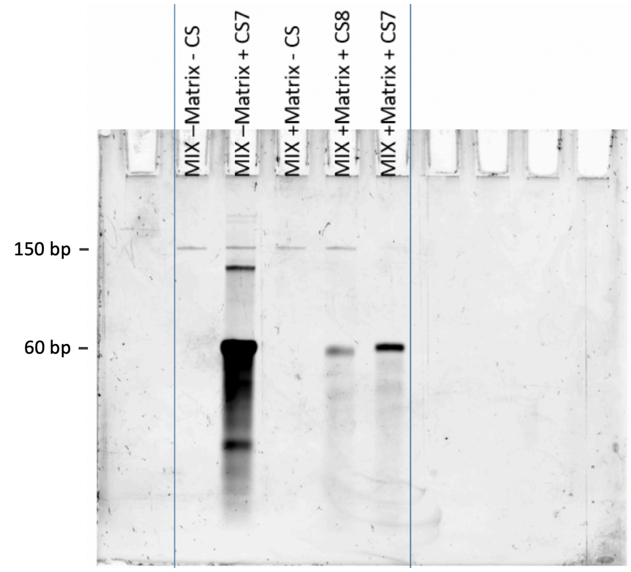

Supplementary Figure 12. *Original uncropped scan of Sybr-Gold-stained dPAGE corresponding to Figure 4b. MIX = dsDNA test target (Sequence: ATGAAAAGCATTTACTTT GTGGCTGGATTATTTGTAATGCTGGTACAAGGCAGCTGGCAACGTTCCCTTCAAGACACAGAGGAGAAATC CAGATCATTCTCAGCTTCCCAGGCAGACCCACTCAGTGATCCTGATCAGATGAACGAGGAC); CS = Catcher strands. CS7 = target-specific catcher strand library (Sequence: see Supplementary Table 3, GCG catcher strands). CS8 = dummy (negative control) catcher strand library (Sequence: see Supplementary Table 3, INS catcher strands).*

Supplementary Tables

Supplementary Table 1. *AF4-LS characterization of **MeRPy-10** and **MeRPy-100**.*

	M_W*1 (10^6 g mol^{-1})	Đ*1 (M_w/M_n)	R_g*2 (nm)	R_h*2 (nm)	ν*2	R_g/R_h *2	$\rho_{app,h}$ (g/L)	$V_{app,h}$ (10^{-3} µm^3)
**MeRPy-10**	5.73	1.80	160	86	0.38	1.86	4.26	2. 7
**MeRPy-10***3	7.43	1.47	173	93	0.39	1.86	4.08	3.4
**MeRPy-100**	8.47	2.24	150	130	0.32	1.15	1.27	9.2

*1 Calculated by dn/dc = 0.160 mL g^{-1}, dn/dc for poly acrylamide was determined (0.156 ml g^{-1}), and extrapolated for MeRPy with dn/dc for DNA (0.168 ml g^{-1})

*2 Main fraction with high data accuracy

*3 Synthesized with 0.002 wt% (instead of 0.005 wt%) APS and TEMED.

M_W = weight average molecular weight, Đ = dispersity index, R_g = radius of gyration, R_h = hydrodynamic radius, ν= scaling factor (according to Eq. 3), $\rho_{app,h}$ = apparent density calculated by R_h, $V_{app,h}$ = apparent volume calculated by R_h.

Supplementary Table 2. *Single-stranded DNA target, catcher and release strands. Red: adapter site; blue: target binding site; green: release site.*

#	Targets	Length [nt]
1	TGTAACATCTGCTGGATCAT	20
2	TGTGCGGACTGGAATGCAAATGAACTGGAT	30
3	GCAAGGATCAAGGTAAAGCTCAGTATAGTCAACGTCAATT	40
4	TCGGAAGTGCTGGGATAAACGGAATGGCCTGAATAGTCGAAGTCAAGATC	50
5	TGCATATCCAGAAGTTCAGTACCCGATCACAGAGTTAGACCATTAGACCATAGCCTTTAC	60
6	TGGACACTGGGATACGAACTTACGAACTTGATCTGATTCTAACTGCCTATAACGAAACTCTATGATAAAA	70
7	TGGTCACGGGTCTCTAAGGTCATAAGTTTCATACGAGGTCAAGGTCAAAGTAGCAATTCCAAGTCAGCGATAAAGTACAA	80
8	CGGTTCATCAAGGTATCAAAGACATCAACTGCCACGTACATAAGCTCGCTCCACTAAGATCCCATTCGTTTAACTAGACC GCTCCAAATC	90
9	GGGATGATGCTGTCGAGCTGGACTCTCACTGTGAACTGAGTTTGGCGTTGCGTTGCGATTATGATCACTCCAATGCATTT TTGCATTGGAGTGATCATAATCGCAACGCAACGCCTTTTGTGCATACAGGTATATCGT	138
10	CGGAGCCGTGCCGCACCGTTCTGTCAGAGTGAGTCAAGCTTTTGGCCCGGCACCCTGCCGCACAATGCCAGCCGTGGTTT ATCAGGCCCAAATAGGTGATATATTTTTATATCACCTATTTGGGCCTGATAAACCACGGCTGGCATTGTGCGGCAGGGTG CCGGGCCTTTGCTCGGAGCTGCCGTCGCCC	190

#	Catcher strands	Length [nt]
1	CCCTTAGCCACGATATGATCCAGCAGATGTTACAATTAGAGCCTTATGAGCCGT	54
2	CCGTTTTAGATCAGTTTGCATTCCAGTCCGCACAATTAGAGCCTTATGAGCCGT	54
3	ATCTTTGTACACTTAGCTTTACCTTGATCCTTGCATTAGAGCCTTATGAGCCGT	54
4	GACAAGCCTTACCCGTTTATCCCAGCACTTCCGAATTAGAGCCTTATGAGCCGT	54
5	GCTGCTACTTTACGACTGAACTTCTGGATATGCAATTAGAGCCTTATGAGCCGT	54
6	TATCAAGCCGTTAAAGTTCGTATCCCAGTGTCCAATTAGAGCCTTATGAGCCGT	54
7	GTTAGCCAAATGAGACCTTAGAGACCCGTGACCAATTAGAGCCTTATGAGCCGT	54
8	ACCCTGCACTTGACTTTGATACCTTGATGAACCGATTAGAGCCTTATGAGCCGT	54
9	TATTTCAGTTTAGTCAGCTCGACAGCATCATCCCATTAGAGCCTTATGAGCCGT	54
10	TACCTACTGACCCTAACGGTGCGGCACGGCTCCGATTAGAGCCTTATGAGCCGT	54

#	Release strands	Length [nt]
1	TGTAACATCTGCTGGATCATATCGTGGCTAAGGG	34
2	TGTGCGGACTGGAATGCAAACTGATCTAAAACGG	34
3	GCAAGGATCAAGGTAAAGCTAAGTGTACAAAGAT	34
4	TCGGAAGTGCTGGGATAAACGGGTAAGGCTTGTC	34
5	TGCATATCCAGAAGTTCAGTCGTAAAGTAGCAGC	34
6	TGGACACTGGGATACGAACTTTAACGGCTTGATA	34
7	TGGTCACGGGTCTCTAAGGTCTCATTTGGCTAAC	34
8	CGGTTCATCAAGGTATCAAAGTCAAGTGCAGGGT	34
9	GGGATGATGCTGTCGAGCTGACTAAACTGAAATA	34
10	CGGAGCCGTGCCGCACCGTTAGGGTCAGTAGGTA	34

Supplementary Table 3. *Catcher strand library (**CSL**) for the genes INS, TTR and GCG. Red: adapter site; blue: target binding site.*

#	INS catcher Strands	Length [nt]
1	ATGGCCCTGTGGATGCGCCTCCTGCCCCTGCTGGCGCTGATTAGAGCCTTATGAGCCGTC	60
2	TGAACCAACACCTGTGCGGCTCACACCTGGTGGAAGCTGATTAGAGCCTTATGAGCCGTC	60
3	ACCCAAGACCCGCCGGGAGGCAGAGGACCTGCAGGTGGGATTAGAGCCTTATGAGCCGTC	60
4	CTGCAGCCCTTGGCCCTGGAGGGGTCCCTGCAGAAGCGGATTAGAGCCTTATGAGCCGTC	60
5	CAAAGGCTGCGGCTGGGTCAGGTCCCCAGAGGGCCAGCGATTAGAGCCTTATGAGCCGTC	60
6	GTGTAGAAGAAGCCTCGTTCCCCGCACACTAGGTAGAGGATTAGAGCCTTATGAGCCGTC	60
7	GCTGCCTGCACCAGGGCCCCCGCCCAGCTCCACCTGCCGATTAGAGCCTTATGAGCCGTC	60
8	GGGAGCAGATGCTGGTACAGCATTGTTCCACAATGCCAGATTAGAGCCTTATGAGCCGTC	60
9	GTTCAAGGGCTTTATTCCATCTCTCTCGGTGCAGGAGGGATTAGAGCCTTATGAGCCGTC	60

#	GCG catcher strands	Length
1	ATGAAAAGCATTTACTTTGTGGCTGGATTATTTGTAATGATTAGAGCCTTATGAGCCGTC	60
2	CAGAGGAGAAATCCAGATCATTCTCAGCTTCCCAGGCAGATTAGAGCCTTATGAGCCGTC	60
3	GCGCCATTCACAGGGCACATTCACCAGTGACTACAGCAGATTAGAGCCTTATGAGCCGTC	60
4	TGGTTGATGAATACCAAGAGGAACAGGAATAACATTGCGATTAGAGCCTTATGAGCCGTC	60
5	CCTTTACCAGTGATGTAAGTTCTTATTTGGAAGGCCAAGATTAGAGCCTTATGAGCCGTC	60
6	AGGAAGGCGAGATTTCCCAGAAGAGGTCGCCATTGTTGGATTAGAGCCTTATGAGCCGTC	60
7	GATGAGATGAACACCATTCTTGATAATCTTGCCGCCAGGATTAGAGCCTTATGAGCCGTC	60
8	TGTCTTGAAGGGAACGTTGCCAGCTGCCTTGTACCAGCGATTAGAGCCTTATGAGCCGTC	60
9	TTGTCCTCGTTCATCTGATCAGGATCACTGAGTGGGTCGATTAGAGCCTTATGAGCCGTC	60
10	CTGCACAAAATCTTGGGCACGCCTGGAGTCCAGATACTGATTAGAGCCTTATGAGCCGTC	60
11	TCCCTTCAGCATGTCTCTCAAATTCATCGTGACGTTTGGATTAGAGCCTTATGAGCCGTC	60
12	CGGCCTTTCACCAGCCAAGCAATGAATTCCTTGGCAGCGATTAGAGCCTTATGAGCCGTC	60
13	AGAGAAAGAACCATCAGCATGTCTGCGGCCAAGTTCTTGATTAGAGCCTTATGAGCCGTC	60
14	CAGTGATTTTGGTCTGAATCAACCAGTTTATAAAGTCCGATTAGAGCCTTATGAGCCGTC	60

#	TTR catcher strands	Length
1	ATGGCTTCTCATCGTCTGCTCCTCCTCTGCCTTGCTGGGATTAGAGCCTTATGAGCCGTC	60
2	GTGAATCCAAGTGTCCTCTGATGGTCAAAGTTCTAGATGATTAGAGCCTTATGAGCCGTC	60
3	TGTGTTCAGAAAGGCTGCTGATGACACCTGGGAGCCATGATTAGAGCCTTATGAGCCGTC	60
4	GGGCTCACAACTGAGGAGGAATTTGTAGAAGGGATATAGATTAGAGCCTTATGAGCCGTC	60
5	CGGTGCCCGTAGGGCCAGCCTCAGACACAAATACCAGTGATTAGAGCCTTATGAGCCGTC	60
6	TGCACGGCCACATTGATGGCAGGACTGCCTCGGACAGCGATTAGAGCCTTATGAGCCGTC	60
7	ATGCAGCTCTCCAGACTCACTGGTTTTCCCAGAGGCAAGATTAGAGCCTTATGAGCCGTC	60
8	GTGCCTTCCAGTAAGATTTGGTGTCTATTTCCACTTTGGATTAGAGCCTTATGAGCCGTC	60

#	Dummy adapter sequence	Length
1	TTTTCGCCAAGTAACTCTTTCCGGTTGTTTGCCCGCCAGATTAGAGCCTTATGAGCCGTC	60

Supplementary Table 4. *Materials and reagents costs for synthesis and purification of **MeRPy-10** and **MeRPy-100** (40–50 mg scale).*

Materials/reagents	Unit	Unit cost	Cost per prep. MeRPy-10	Cost per prep. MeRPy-100
Anchor strand	1 nmol	$0.20	$16.08	$160.76
Acrylamide	1 g	$0.36	$0.01	$0.01
Sodium acrylate	1 g	$2.42	$0.00	$0.00
APS	1 g	$0.42	$0.00	$0.00
TEMED	1 mL	$1.53	$0.00	$0.00
N_2 gas, purified	1 m^3	$1.50	$0.03	$0.03
1 ml syringe	1	$0.15	$0.15	$0.15
2 ml glass vial	1	$0.49	$0.49	$0.49
22G syringe needle	1	$0.10	$0.10	$0.10
TBE buffer, 5x	1 mL	$0.06	$0.01	$0.01
TE buffer, 100x	1 mL	$0.45	$0.09	$0.09
1 ml pipette tips	1	$0.03	$0.25	$0.25
250 µL pipette tips	1	$0.03	$0.27	$0.27
20 µL pipette tips	1	$0.03	$0.55	$0.55
PCR tube	1	$0.05	$0.30	$0.30
NaCl, 5M	1 mL	$0.02	$0.00	$0.01
Methanol	1 L	$6.07	$0.09	$0.09
15 ml Falcon tube	1	$0.15	$0.30	$0.30
**Total cost**			**$18.72**	**$163.41**
**Cost per nmol binding capacity**			**$0.31**	**$0.27**

Supplementary Table 5. *Anchor strand.*

#	Anchor strand	Length [nt]
1	/5Acryd/GACGGCTCATAAGGCTCTAAXC	20–22

Underlined bases were absent in an early MeRPy synthesis. X = T or U (deoxy-uridine)

Supplementary References

1. Boye, S., Polikarpov, N., Appelhans, D. & Lederer, A. An alternative route to dye-polymer complexation study using asymmetrical flow field-flow fractionation. *J. Chromatogr. A* **1217**, 4841–4849 (2010).
2. Ennen, F. *et al.* Biohybrid structures consisting of biotinylated glycodendrimers and proteins: influence of the biotin ligand's number and chemical nature on the biotin–avidin conjugation. *Polym. Chem.* **5**, 1323–1339 (2014).
3. Boye, S. *et al.* From 1D Rods to 3D Networks: A Biohybrid Topological Diversity Investigated by Asymmetrical Flow Field-Flow Fractionation. *Macromolecules* **48**, 4607–4619 (2015).
4. Gumz, H. *et al.* Toward Functional Synthetic Cells: In-Depth Study of Nanoparticle and Enzyme Diffusion through a Cross-Linked Polymersome Membrane. *Adv. Sci.* **6**, 1801299 (2019).
