# Supplementary material for: A smart polymer for sequence-selective binding, pulldown, and release of DNA targets
Source: Commun Biol. 2020 Jul 10;3:369. doi: 10.1038/s42003-020-1082-2 (PMC7351716; doi:10.1038/s42003-020-1082-2)
Supplement: Supplementary file 2 — Description of Additional Supplementary Files [file 42003_2020_1082_MOESM2_ESM.pdf]

Description of Additional Supplementary Files

File Name: Supplementary Data 1
Description: Generator script for catcher strand library (Python source code)

File Name: Supplementary Data 2
Description: Generator script for catcher strand library (compiled for Windows).

File Name: Supplementary Data 3
Description: Generator script for catcher strand library (compiled for MacOS).

File Name: Supplementary Data 4
Description: Raw data underlying Figure 1c

File Name: Supplementary Data 5
Description: Raw data underlying Figure 3b.
