# Supplementary material for: A smart polymer for sequence-selective binding, pulldown, and release of DNA targets
Source: Commun Biol. 2020 Jul 10;3:369. doi: 10.1038/s42003-020-1082-2 (PMC7351716; doi:10.1038/s42003-020-1082-2)
Supplement: Supplementary file 9 — Reporting Summary [file 42003_2020_1082_MOESM9_ESM.pdf]

nature research

Corresponding author(s): Elisha Krieg, William M. Shih

Last updated by author(s): 03.06.20

Reporting Summary

Nature Research wishes to improve the reproducibility of the work that we publish. This form provides structure for consistency and transparency in reporting. For further information on Nature Research policies, see Authors & Referees and the Editorial Policy Checklist .

Statistics

For all statistical analyses, confirm that the following items are present in the figure legend, table legend, main text, or Methods section.

n/a	Confirmed	
☐	☒	The exact sample size (*n*) for each experimental group/condition, given as a discrete number and unit of measurement
☐	☒	A statement on whether measurements were taken from distinct samples or whether the same sample was measured repeatedly
☐	☒	The statistical test(s) used AND whether they are one- or two-sided<br>*Only common tests should be described solely by name; describe more complex techniques in the Methods section.*
☒	☐	A description of all covariates tested
☒	☐	A description of any assumptions or corrections, such as tests of normality and adjustment for multiple comparisons
☐	☒	A full description of the statistical parameters including central tendency (e.g. means) or other basic estimates (e.g. regression coefficient) AND variation (e.g. standard deviation) or associated estimates of uncertainty (e.g. confidence intervals)
☐	☒	For null hypothesis testing, the test statistic (e.g. *F*, *t*, *r*) with confidence intervals, effect sizes, degrees of freedom and *P* value noted<br>*Give P values as exact values whenever suitable.*
☒	☐	For Bayesian analysis, information on the choice of priors and Markov chain Monte Carlo settings
☒	☐	For hierarchical and complex designs, identification of the appropriate level for tests and full reporting of outcomes
☒	☐	Estimates of effect sizes (e.g. Cohen's *d*, Pearson's *r*), indicating how they were calculated

Our web collection on statistics for biologists contains articles on many of the points above.

Software and code

Policy information about availability of computer code

Data collection	Fluorescence imaging was performed on a Typhoon Scanner with the software Image Reader (v1.0). PCR and qPCR were performed with the CFX Manager (v3.0). TPM values were generated with Kallisto (v0.43.0).
Data analysis	Standard data analysis and plotting was performed in Microsoft Excel for Mac (v16.16.21) and Origin 2018 (64 bit). Analysis of fluorescent images was performed in FIJI (v1.0). TPM plots and heatmaps of correlation matrices were generated in R software (v.3.6.1), using ggplot2 and pheatmap packages. The coverage plots were generated by aligning each library and calculating the coverage using bedtools genomecov (v2.27.1) package on Python 3.7 and plotting them on R.

For manuscripts utilizing custom algorithms or software that are central to the research but not yet described in published literature, software must be made available to editors/reviewers. We strongly encourage code deposition in a community repository (e.g. GitHub). See the Nature Research guidelines for submitting code & software for further information.

Data

Policy information about availability of data

All manuscripts must include a data availability statement. This statement should provide the following information, where applicable:

- Accession codes, unique identifiers, or web links for publicly available datasets
- A list of figures that have associated raw data
- A description of any restrictions on data availability

Gene expression profile data is publicly available at the GEO database (accession number: GSE150165). All other raw data generated during and/or analyzed during the current study are available from the corresponding authors on reasonable request.

Field-specific reporting

Please select the one below that is the best fit for your research. If you are not sure, read the appropriate sections before making your selection.

- [x] Life sciences
- [ ] Behavioural & social sciences
- [ ] Ecological, evolutionary & environmental sciences

For a reference copy of the document with all sections, see nature.com/documents/nr-reporting-summary-flat.pdf

Life sciences study design

All studies must disclose on these points even when the disclosure is negative.

Sample size	Sample sizes are noted in the figure captions and methods section. Illumina sequencing was typically performed with at least three independent replicates for each condition. For inexpensive methods (e.g. gel electrophoresis), sample size was chosen to be at least n = 10 to obtain average values and standard deviations.
Data exclusions	No data were excluded from the analysis.
Replication	All experiments were performed multiple times and showed consistent results, as reported in the manuscript.
Randomization	This study did not contain experiments that required randomized allocation into experimental groups.
Blinding	Blinding was not necessary in this study, as all obtained data is based on quantitative analytical chemistry and molecular biology techniques.

Reporting for specific materials, systems and methods

We require information from authors about some types of materials, experimental systems and methods used in many studies. Here, indicate whether each material, system or method listed is relevant to your study. If you are not sure if a list item applies to your research, read the appropriate section before selecting a response.

Materials & experimental systems

n/a	Involved in the study	
[x]	[ ]	Antibodies
[x]	[ ]	Eukaryotic cell lines
[x]	[ ]	Palaeontology
[x]	[ ]	Animals and other organisms
[ ]	[x]	Human research participants
[x]	[ ]	Clinical data

Methods

n/a	Involved in the study	
[x]	[ ]	ChIP-seq
[x]	[ ]	Flow cytometry
[x]	[ ]	MRI-based neuroimaging

Human research participants

Policy information about studies involving human research participants

Population characteristics	cDNA libraries for INS, GCG and TTR pulldown were generated from pancreatic islets obtained from a single individual. The particular cDNA library samples were chosen due to their availability in sufficient quantities, allowing sufficient numbers of replicate and control experiments. Pancreatic cDNA libraries were chosen due to the high abundance of a few genes (INS, GCG, TTR), where abundant transcript depletion is a particularly effective enhancement strategy. Patient-specific information cannot be disclosed and is irrelevant to the findings in this study.
Recruitment	There was no dedicated patient recruitment for this study.
Ethics oversight	Medical School of the Technical University of Dresden

Note that full information on the approval of the study protocol must also be provided in the manuscript.
